# Sugar-free Workplace: A Step for Fighting Obesity

**DOI:** 10.7759/cureus.6336

**Published:** 2019-12-10

**Authors:** Sahand Bamarni, Dereen Mohammed Saeed, Subhasis Misra

**Affiliations:** 1 Surgery, Brandon Regional Hospital, Brandon, USA; 2 Pathology, University of Illinois, Chicago, USA

**Keywords:** sugar free, sugar, obesity, pandemic

## Abstract

Currently, there is a worldwide obesity pandemic with an incidence that has increased progressively over the last few decades. Obesity is considered a global health hazard and is associated with a significant economic impact on the healthcare system. It has been linked to several serious medical conditions, including heart disease, hypertension, stroke, diabetes mellitus, and cancer. Obesity is also related to social and psychological problems such as anxiety and depression. Several factors predispose the population to obesity, including decreased physical activity and non-healthy dietary habits. Sugar is the most important key contributor to the pandemic of obesity, and implementing a sugar-free workplace policy will provide a promising strategy for fighting obesity.

## Introduction and background

**Figure 1:**
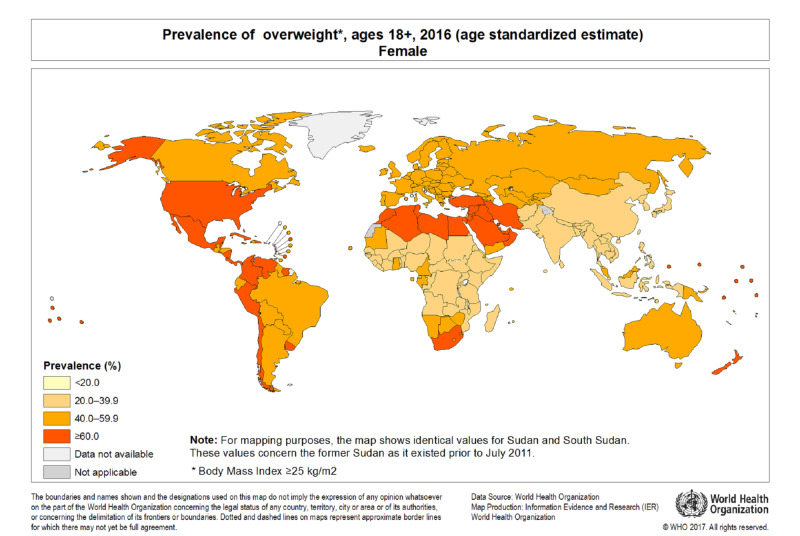
WHO world map of prevalence of overweight in adult female.

**Figure 2:**
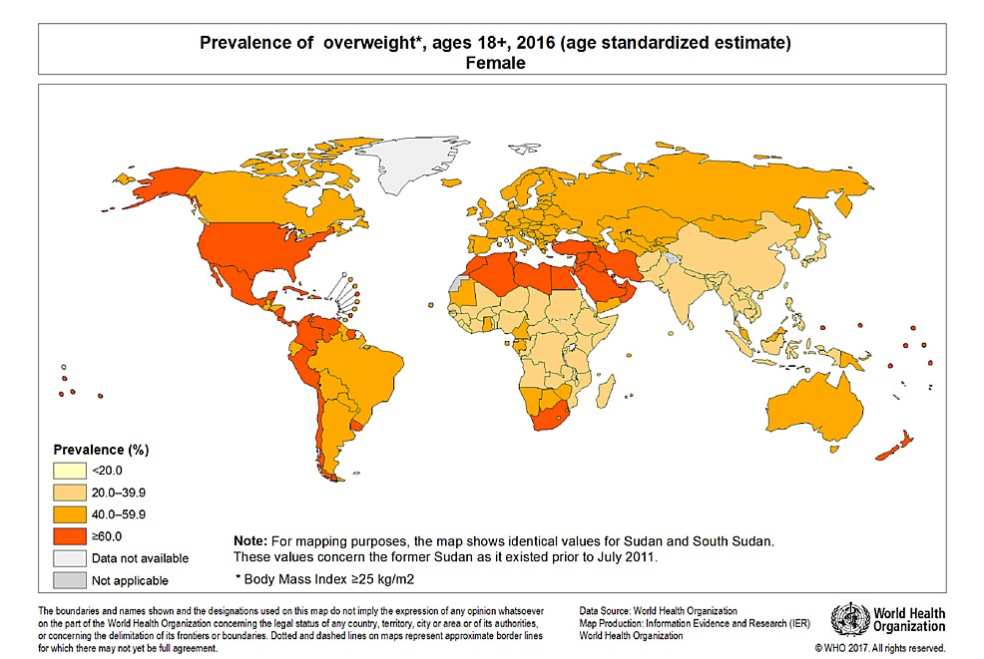
WHO world map of prevalence of overweight in adult male.

Obesity is regarded as a global health problem (Figures 1, 2), and it has surpassed malnutrition in both prevalence and related deaths. There is presently a worldwide pandemic of obesity-a change from being an epidemic health problem primarily in high-income countries. The prevalence of obesity has tripled over the last four decades, with an estimated 13% of the world’s adult population being obese in 2016 and almost 40% being overweight. While obesity prevalence is increasing in adults, there has been a more dramatic increase in pediatric populations, with the prevalence increasing by 4.5 times since 1975, and more than 380 million children being overweight or obese in 2016 [1].

**Figure 3:**
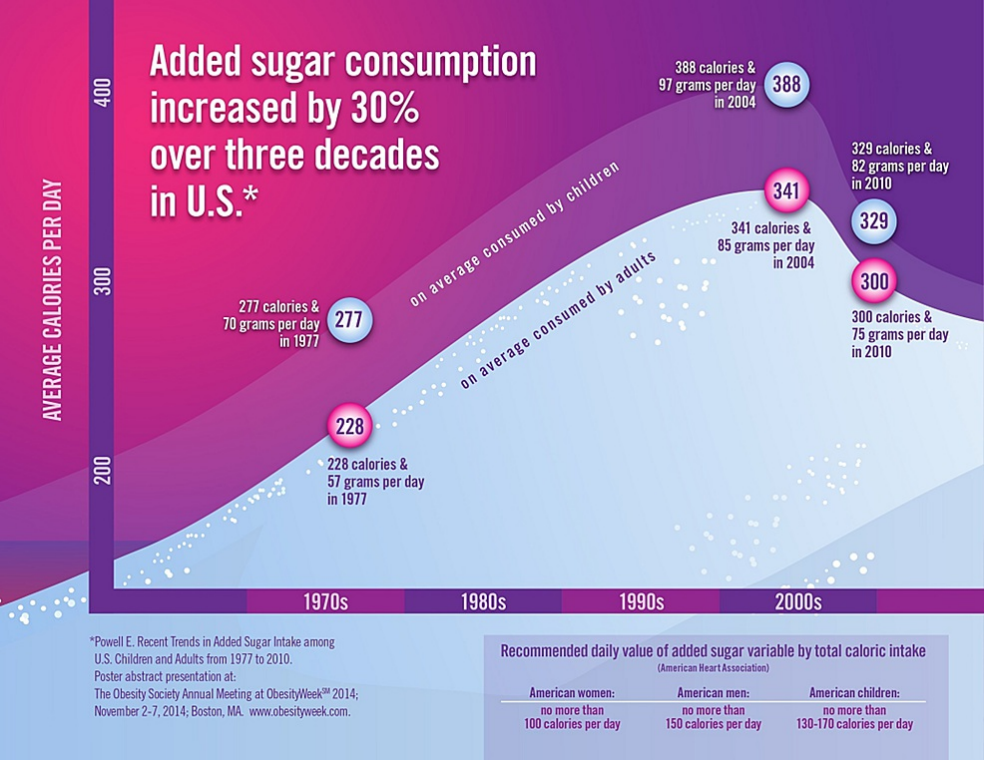
Added sugar consumption in the United States from 1977 to 2010.

Obesity in the United States is a much more common health problem than any other health issue, with a prevalence of about 40% [2]. It is estimated that the annual cost of obesity was $147 billion in 2008 [2]. Global obesity contributed to four million deaths and 120 million disability-adjusted life years (DALYs). Obesity-related mortality rate increased by 28% from 41.9 per 100,000 in 1990 to 53.7 per 100,000 in 2015. Similarly, obesity-related DALYs increased by 35.8% from 1,200 per 100,000 in 1990 to 1,630 per 100,000 in 2015 [3]. An increase in the prevalence of obesity is attributed to the increased consumption of added sugars. It is estimated that more than 55% of people in the United States consumes over 50 grams added sugar daily (Figure 3), which is considered more than the advised maximum daily intake according to American Heart Association. World Health Organization (WHO) recent guideline recommends adults and children reduce their daily intake of free sugars to less than 10% of their total energy intake. A further reduction to below 5% or roughly 25 grams (six teaspoons) per day would provide additional health benefits [4]. 

## Review

Dietary factors are considered to be the major drivers for obesity. Increased availability and easy access to unhealthy food contribute to the global obesity. Total energy intake increased over the last five decades for all income groups. From 1965 to 2008, the total energy intake increased by 634, 462, and 492 KJ/day in low, middle, and high socioeconomic groups, respectively [5]. Increased energy consumption was attributed to the increased frequency of eating away from home foods. It is estimated that home consumed diet decreased by 23% from 1965 to 2008 [5]. National Center for Health Statistics in 2013 published data on consumption of added sugar between 2005 and 2010 in the United States with approximately 14% of young adults' total caloric intakes obtained from added sugars during this period [6].

The key targets for any obesity control policy are the food and physical activity environments. However, reduced physical activities are considered as potential factors for global obesity but are less likely to be major contributors since urbanization with subsequent decline in the physical activities preceded the obesity pandemic [4]. Different interventions are usually suggested by healthcare providers to promote increased physical activity and a healthy diet. Unfortunately, nearly half of patients are not very engaged or not engaged at all in addressing their obesity [7]. It is of critical importance to implement people-based strategies for the prevention of obesity that target factors at both personal and environmental levels. These strategies aim to alter physical activity environments to facilitate higher levels of physical activities and to reduce sedentary lifestyles. Additionally, they provide healthier food choices through different food policies (e.g., nutritional labeling, formulation, restricting unhealthy food, and providing healthier choices at lower prices). It has been shown that reducing the price by half on healthier food items will increase their purchase by 93% [8].

Food landscape

The “food landscape” represent what, when, where, and how the food is presented. Easy access and availability of unhealthy food with intense food marketing and advertisement are the two major factors contributing to unhealthy dietary habits with subsequent weight gain. Resolving this crisis may require focusing on both individual behavior and food environments that serve as barriers to nutritional behavior change. While individual measures are not always effective in controlling obesity, improving food environments and marketing serve as barriers to unhealthy nutritional behavior.

The rationale

Workplaces play a critical role in raising awareness of obesity and an unhealthy diet and can make a greater impact on the community's eating habits; therefore, implementing a policy or intervention to reduce obesity in the workplace setting is an ideal situation. Unfortunately, the prevalence of obesity among healthcare workers is very concerning, with no significant difference in the prevalence of obesity between nurses and people working in non-healthcare-related jobs. At the same time, obesity among healthcare professionals has a negative impact on a personal level and may negatively promote obesity [9]. Evidence for reducing weight is promising for worksite programs; however, the effects on other cardiometabolic risk factors are inconsistent [10]. For any obesity control intervention to be effective, it should be a policy and not just a promotional effort. The best example of this is seen in the results of the Step Ahead trial, a randomized controlled trial involving six hospitals in Massachusetts [11]. The trial combined ecologic interventions to prevent weight gain of hospital employees with strategies to promote physical activity (e.g., stairway signs, outdoor walking routes) and healthy eating, as well as campaigns and challenges for weight loss, physical activity, and healthy eating. No significant differences in body mass index at one- and two-year follow-ups were demonstrated [11]. Participation in this trial was low, and likely there was no consistent adherence to all interventions, which could be attributed to the level of medical knowledge and education of the participants. Hospitals, like other workplaces, have a high prevalence of obesity.

Policy implication

Sugar-sweetened drinks and diet may be key contributors to the epidemic of obesity [12]. These drinks and food provide high sugar with low satiety [12]. A systematic review and meta-analysis of 30 trials and 38 prospective cohort studies showed a significant association between sugar and obesity in adults [13]. Another systematic review and meta-analysis of 22 prospective cohort studies and randomized controlled trials provides evidence that consumption of sugar-sweetened beverages (SSB) is associated with weight gain in children and adults [14]. SSB consumption is also associated with increased incidence of diabetes mellitus (DM) [15], metabolic syndrome [16], hypertension [17], coronary heart disease [18,19], stroke [20], and gout [21]. School-based interventions demonstrate a lower incidence of obesity among students with limited soft drink consumption [12]. It has been shown that replacing soft drinks with healthier choices in vending machines could actually increase sales while significantly reducing calorie intake per drink purchased [22]. Many hospitals across the country implement the sugar-free hospital policy. Participating hospitals include Baylor Health Care System (Dallas, Texas), Cleveland Clinic Foundation (Cleveland, Ohio), Indiana University Health (Indianapolis, Indiana), Children’s Mercy Hospital (Kansas City, Missouri), Seattle Children’s Hospital (Seattle, Washington), University of Michigan Health System (Ann Arbor, Michigan), and several hospitals in Minnesota and Wisconsin through the Commons Healthcare Challenge program [23].

Health and economic impact

Reducing sugar consumption likely will have a major positive impact at both the health and economic levels. Consumption of supraphysiological dosage of sugar has been associated with increased risk for coronary artery disease [24], hyperlipidemia [25,26], hypertension [27-29], DM [30-32], non-alcoholic fatty liver disease [33,34], and cancer [35,36]. Vreman et al. estimated that a 20% reduction in added sugars intake will effectively reduce the prevalence of coronary artery disease, DM, fatty liver, and obesity [37]. The Diabetes Remission Clinical Trial (DiRECT), a recent randomized controlled trial on type 2 DM, demonstrated that using intensive weight management and restricted diet program will cause remission of type 2 DM in almost half of the patients. A mean weight loss of 10 kg with more than 15 kg weight loss in 24% after one year of intervention was reported [38]. The economic benefits are mainly obtained by reducing the cost of metabolic disease and obesity-related health problems. A simulated open cohort study is being conducted from 2015 to 2035, with a base cohort of more than 22,000 patients with new people entering the model each year at age 20 [37]. The study aims to demonstrate that an estimated reduction of 20% of sugar will reduce annual direct medical costs for US adults by more than $10 billion by the year 2035, while a 50% reduction will save $21 billion. In addition, health outcomes will significantly improve with 20% reduction of sugar consumption by averting about 770,000 DALYs, while 50% reduction in consumption will avert another 1.6 million DALYs [37].

Limitations

Energy imbalance is a major determinant of the potential for dietary sugars to influence weight changes. Sugar consumption should not be the sole determinant of a healthy diet since there are many other dietary factors contributing to obesity like consumption of excess calories and high fat diet. It would be a difficult goal to legislate and modify individual's eating behavior which both may play major roles in limiting the implementation of a dietary policy.

## Conclusions

It is important to identify the determinants of the surge in national and global obesity rates and to implement sugar-free policy interventions in the workplace to control the pandemic of obesity and metabolic syndrome. Workplace cafeterias, cafes, vending machines, and gift shops should offer healthy meals with more fruits, vegetables, and low-fat diet and sugar-free drinks.
